# Robust B-exciton emission at room temperature in few-layers of MoS_2_:Ag nanoheterojunctions embedded into a glass matrix

**DOI:** 10.1038/s41598-020-72899-3

**Published:** 2020-09-24

**Authors:** Abdus Salam Sarkar, Ioannis Konidakis, Ioanna Demeridou, Efthymis Serpetzoglou, George Kioseoglou, Emmanuel Stratakis

**Affiliations:** 1 Institute of Electronic Structure and Laser, Foundation for Research and Technology-Hellas, 700 13 Heraklion, Crete Greece; 2 Physics Department, University of Crete, 710 03 Heraklion, Crete Greece; 3 Department of Materials Science and Technology, University of Crete, 710 03 Heraklion, Crete Greece

**Keywords:** Engineering, Materials science, Nanoscience and technology

## Abstract

Tailoring the photoluminescence (PL) properties in two-dimensional (2D) molybdenum disulfide (MoS_2_) crystals using external factors is critical for its use in valleytronic, nanophotonic and optoelectronic applications. Although significant effort has been devoted towards enhancing or manipulating the excitonic emission in MoS_2_ monolayers, the excitonic emission in few-layers MoS_2_ has been largely unexplored. Here, we put forward a novel nano-heterojunction system, prepared with a non-lithographic process, to enhance and control such emission. It is based on the incorporation of few-layers MoS_2_ into a plasmonic silver metaphosphate glass (AgPO_3_) matrix. It is shown that, apart from the enhancement of the emission of both A- and B-excitons, the B-excitonic emission dominates the PL intensity. In particular, we observe an almost six-fold enhancement of the B-exciton emission, compared to control MoS_2_ samples. This enhanced PL at room temperature is attributed to an enhanced exciton–plasmon coupling and it is supported by ultrafast time-resolved spectroscopy that reveals plasmon-enhanced electron transfer that takes place in Ag nanoparticles-MoS_2_ nanoheterojunctions. Our results provide a great avenue to tailor the emission properties of few-layers MoS_2_, which could find application in emerging valleytronic devices working with B excitons.

## Introduction

Two-dimensional (2D) Transition Metal Dichalcogenides (TMDs) provide an appealing platform for emerging atomic scale research in nanophotonic and optoelectronic applications^1–4^. Monolayer molybdenum disulfide (MoS_2_), in particular, gains considerable attention due to its direct band gap and potential integration with other nanostructures to form nanoscale van der Waals heterojunctions with intriguing physical and optical properties^5^. Indeed, it has been shown that the optical properties of MoS_2_ monolayers, such as photoluminescence (PL), can be manipulated through its coupling with nanomaterials of various dimensionalities. In particular, zero dimensional (0D) quantum dots and nanoparticles^6,7^, one-dimensional (1D) nanowires and nanorods^8–10^, as well as other 2D materials^5,11^ had been combined with monolayer MoS_2_ to manipulate its emission intensity and/or quantum yield. Besides this, polymeric spacing^12^, defect engineering^13^, doping^14^, and chemical modification^15^ approaches were employed to manipulate the emission properties. However, monolayer MoS_2_ suffers from low intrinsic photoluminescence (PL) quantum yield (0.01–0.6%), dominated by the A-excitonic emission, due to its sub nanometer thickness and defect density mediated nonradiated recombination^1^. The low PL yield was overcome (more than 95%) with chemical treatment by an organic superacid^16^. In contrast to a monolayer MoS_2_, few layers of MoS_2_ have several orders of magnitude lower PL quantum yield^1^. On the other hand, few-layers MoS_2,_ as an indirect semiconductor, have significantly larger optical density, which enhances its external quantum efficiency^17^. Owing to this advantage, research on the PL properties in few layers MoS_2_ has received significant attention. For example, metallic and other nanostructures^5,7^ were used to manipulate the A-excitonic emission in few layers of MoS_2_^18^. However, this approach has only been limited to the enhancement of the A-excitonic emission.

On the other hand, transparent thermoplastic glasses (TTG) were extensively used for homogeneous incorporation of 2D layered materials. However, the relevant studies were limited to measure the nonlinear optical response of the embedded 2D nanoflakes^19,20^. On a rather different manner photonic crystal cavities^21–23^, as well as Mie-resonant metasurfaces^6^, have been employed to tailor the optical properties of MoS_2_. Similar to the case of nanostructures, the manipulation of PL emission has been only limited to A-exciton. Mikkelsen and co-workers^24,25^ were the first who carried out a systematic study to manipulate the B- excitonic emission of a single-layer of MoS_2_. However, the study of the emission properties was limited to the ground A-exciton state. Nevertheless, a detailed investigation of the B-exciton state in the ultrafast regime is crucial to shed light on the physical phenomena that take place.

In this study, we present the development of a nanohybrid heterojunction system composed of few layers of MoS_2_ embedded into a silver metaphosphate glass (AgPO_3_), as a means to enhance and control the MoS_2_ exciton emission. The selection of AgPO_3_ glass as a host matrix is prompted by several reasons: First, its transparency in most of the visible range (Fig. S1) enables the full exploitation of the AgPO_3_:MoS_2_ photoluminescence properties towards various nanophotonic applications^26^. Moreover, the presence of silver nanoparticles (NPs) within the glass matrix gives rise to interesting optical phenomena that can be exploited towards enhancing and manipulating the PL properties of the incorporated MoS_2_ layers. Finally, the AgPO_3_ glass exhibits a very low glass transition temperature of 192 °C, which is indicative of its soft nature. As a consequence, the MoS_2_ integration process is performed at low temperatures, suitable to avoid any oxidation. On top of that, an advanced 2D exciton–plasmon system composed of a few-layer TMD integrated with a semiconducting metal-phosphate glass is realized. It is shown that the layered TMDs create nanoscale van der Waals heterojunctions with the metallic nanostructures of the glass, which can be exploited to tailor light-matter interactions at the nanoscale.

## Results

### Fabrication and characterization of MoS_2_ and nanoheterojunctions

**Figure 1:**
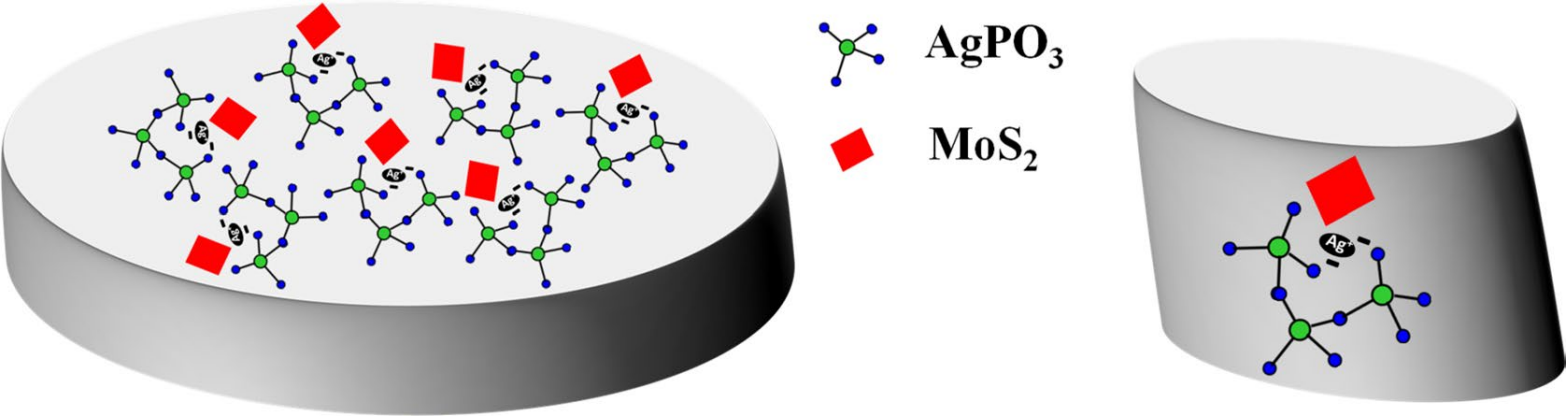
Schematic representation of the composite transparent silver metaphosphate glass (AgPO_3_) matrix incorporating the MoS_2_ flakes. The right panel depicts a single AgPO_3_:MoS_2_ nanoheterojunction.

The MoS_2_ flakes were obtained by liquid exfoliation (see “Methods”)^27^. The lateral dimensions of the MoS_2_ nanoflakes, as determined by SEM imaging, were found to lie within the micrometer range (Fig. S2a), while, the average thickness measured by AFM was ~ 4 nm (Fig. S2b, c). A schematic representation of the composite glass, comprising numerous AgPO_3_:MoS_2_ nano-heterojunctions is illustrated in Fig. 1 (see “Methods”).

### Optical spectroscopy

**Figure 2:**
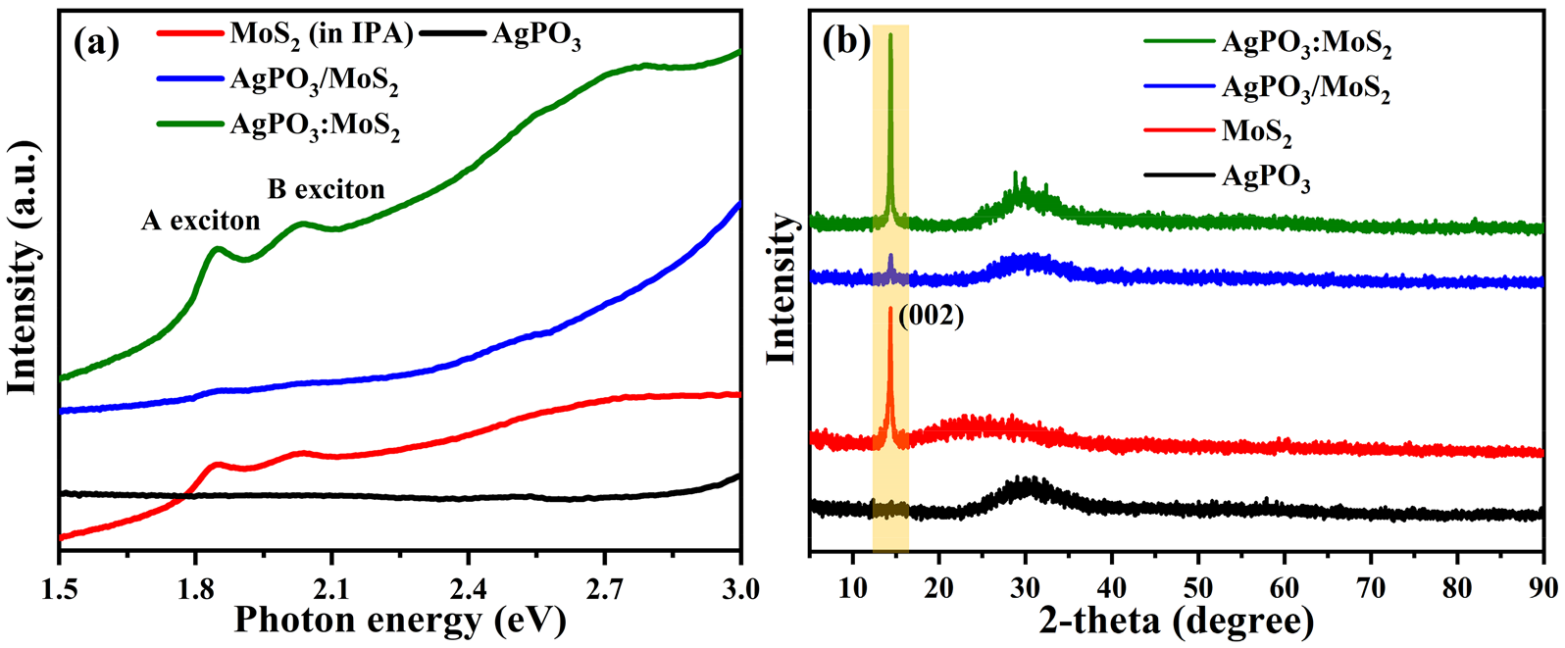
Spectroscopic characterizations of MoS_2_ and their heterojunctions. (**a**) Optical absorption spectra and (**b**) X-ray diffraction pattern, of MoS_2_ (MoS_2_ on glass substrate), AgPO_3_, AgPO_3_/MoS_2_ (MoS_2_ on AgPO_3_), and AgPO_3_:MoS_2_ (MoS_2_ embedded into AgPO_3_).

Absorption spectroscopy was employed to confirm the formation of nano-heterojunctions between MoS_2_ and AgPO_3_. The pristine AgPO_3_ glass exhibits two characteristic peaks at 2.0 and 2.5 eV (Fig. S3a,b), which correspond to the Ag plasmonic bands and are attributed to a bimodal distribution of isolated nanoparticles or their clusters attained due to the phosphate matrix. Another reason of the emergence of the plasmonic band at 2.0 eV is the clustering/agglomeration of Ag NPs observed. Indeed, as the effective nanoparticles size increases, a corresponding red shift in the plasmon band occurs. This is also indicated by the broad plasmonic band centered at 2.0 eV. The absorption spectrum of bare MoS_2_ flakes exhibits the two characteristic excitonic peaks at 1.84 eV (A-exciton) and 2.03 eV (B-exciton) respectively (Fig. 2a, red line)^1^. Both peaks were also present in the absorption spectrum of the AgPO_3_:MoS_2_ heterojunctions of composite matrix (green line of Fig. 2a), i.e. in which the MoS_2_ is incorporated within the glass. At the same time, the absorption intensity of AgPO_3_:MoS_2_, is enhanced compared to the pristine AgPO_3_. The small blue shift of the A- and B-exciton peak positions by 13 meV and 17 meV, respectively, is due to the change of the dielectric environment rather than any oxidation process. The integration of MoS_2_ within AgPO_3_ glass took place at 170 °C, which is below the glass transition (T_G_), and thus hard to cause significant oxidation of the phosphate network. Instead, the modification of the dielectric constant, from that of the solvent to the higher dielectric constant of the surrounding glass matrix, could be the reason for the observed shift of the exciton states. It is noted that these findings are not observed when the MoS_2_ flake is positioned on the surface of AgPO_3_ glass, i.e. AgPO_3_/MoS_2_ spectrum in Fig. 2a (blue line).

It is widely acknowledged that the trigonal prismatic phase (2H-phase) integrity plays a crucial role in PL emission of exfoliated MoS_2_. Aiming to identify the phase integrity in MoS_2_ flakes dispersed into AgPO_3_, a series of structural studies have been carried out. In particular, the X-ray diffraction pattern of MoS_2_ (Fig. 2b) exhibits a strong peak located at 14.34°, corresponding to the (002) plane, which agrees well with the hexagonal MoS_2_^28,29^. Besides this, the examination of the AgPO_3_/MoS_2_ and AgPO_3_:MoS_2_ matrix showed a primary peak at 2θ ~ 14.38°, indicating that the liquid exfoliation of few layers did not change the MoS_2_ structure. Since the peak position in XRD is not changing for both structures (Fig. S3), there is no significant strain induced when MoS_2_ is incorporated into the phosphate matrix. However, a reduction in the full width at half maximum (FWHM) has been observed (Fig. S3c and Table S1) and this could be due to the variation in the microstructure, the grain distortion, and dislocation density of the crystal^30,31^. The possibility that higher crystallinity may have reduce the FWHM of MoS_2_ (because of the heating process) was excluded by performing a controlled experiment in MoS_2_ treated under the same conditions used for the fabrication of AgPO_3_:MoS_2_. No changes in the peak position as well as in the FWHM were observed (Fig. S3b).

**Figure 3:**
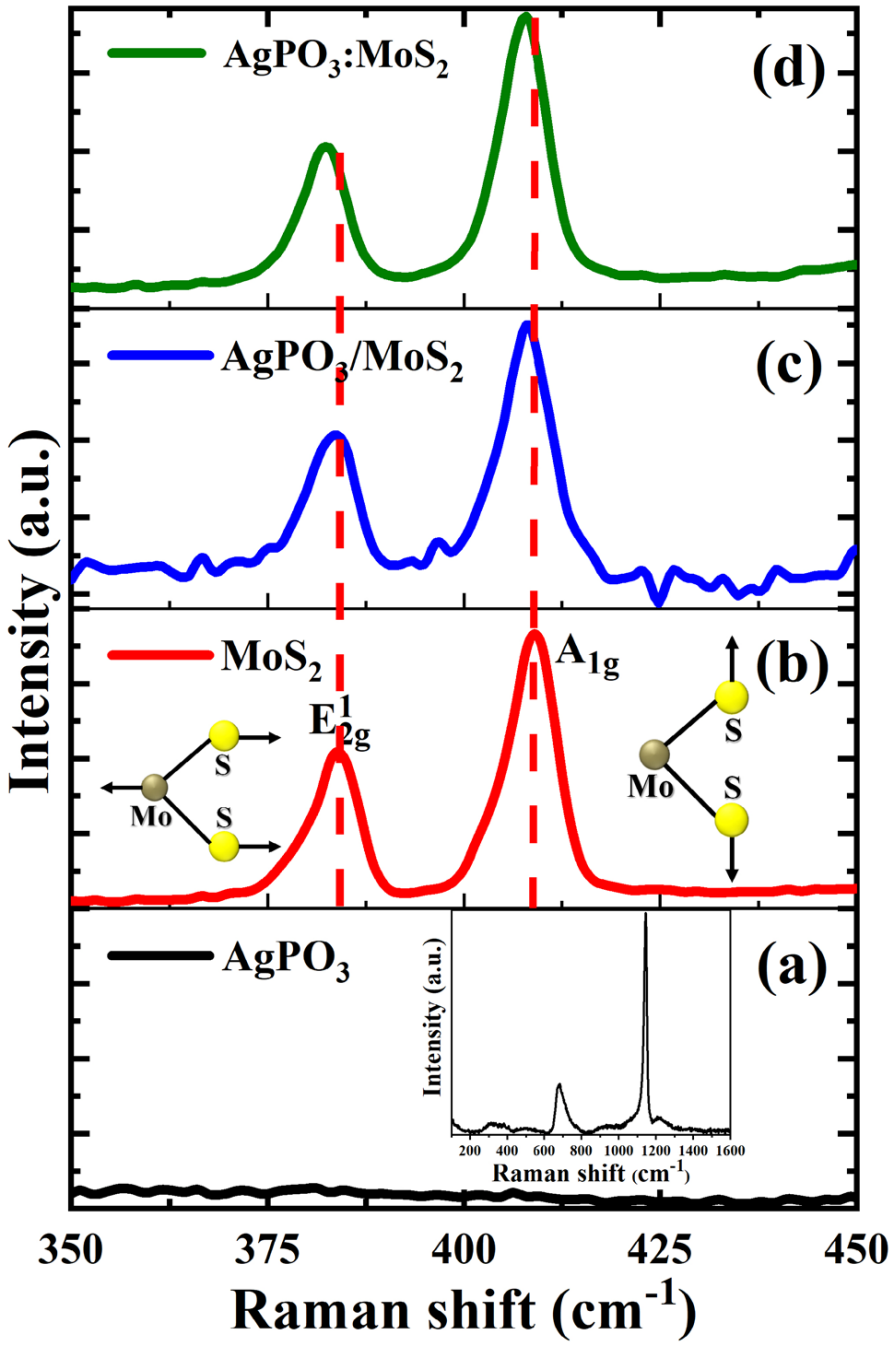
Raman spectra of (**a**) AgPO_3_ glass, (**b**) MoS_2_ flakes on Si (Si/MoS_2_), (**c**) MoS_2_ on AgPO_3_ (AgPO_3_/MoS_2_) and (**d**) MoS_2_ embedded into AgPO_3_ (AgPO_3_:MoS_2_). The inset presents the Raman spectrum of the pristine AgPO_3_ glass in a wide range.

In addition, the Raman spectra (Fig. 3) of AgPO_3_:MoS_2_ composite glass were obtained and compared with that of a bare MoS_2_. The Raman spectra of bare MoS_2_ depict two characteristic peaks at 382.83 and 407.12 cm^−1^, corresponding to the in-plane (${\text{E}}_{{2{\text{g}}}}^{1}$) and out of plane (${\text{A}}_{{1{\text{g}}}}$) vibrational modes. The Raman frequency difference (Δω = ω(${\text{A}}_{{1{\text{g}}}}$)—ω(${\text{E}}_{2g}^{1}$)) between these two modes dependents on the number of layers, which is used to determine the MoS_2_ thickness^32,33^; this difference (Δω) is measured to be 24–25 cm^−1^, indicating that the MoS_2_ flakes have several layers^32–34^. This number is found to be similar for all the samples studied (Fig. S4). Besides this, the full width at half maximum (FWHM) of ${\text{E}}_{2g}^{1}$ is ~ 5.67 cm^−1^, suggests good crystallinity of the exfoliated MoS_2_^35,36^. Furthermore, a small red shift of about 2 cm^−1^ in both Raman modes has been observed in AgPO_3_:MoS_2_^36^. This shift is unlikely to be due to strain since it is the same for both modes and not only for the in-plane one (a signature of induced strain in the system). The inset of Fig. 3 also shows the obtained Raman spectrum of the pristine AgPO_3_ glass, i.e. prior to any MoS_2_ incorporation. The spectrum of AgPO_3_ glass exhibits a major band at around 1142 cm^−1^, whereas a broader band at ~ 675 cm^−1^ is also present. The first Raman signature is attributed to the symmetric stretching vibration of terminal PO_2_^−^ groups, v_s_(PO_2_^−^), while the latter features originates from the symmetric stretching movement of P-O-P bridges within the phosphate backbone, v_s_(P-O-P)^37,38^.

**Figure 4:**
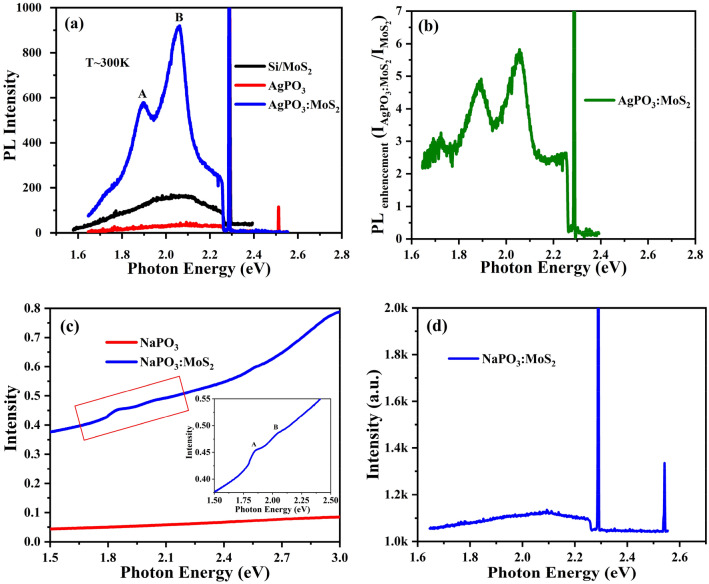
Room temperature spectroscopic characteristics. (**a**) PL emission spectrum of Si/MoS_2_, AgPO_3_, and AgPO_3_:MoS_2_. (**b**) PL intensity ratio of AgPO_3_:MoS_2_ and Si/MoS_2_ samples. (**c**) Absorption spectra of NaPO_3_, and NaPO_3_:MoS_2_ (Inset: Magnified spectra of MoS_2_ excitonic peaks in NaPO_3_:MoS_2_) and (**d**) PL emission spectrum of NaPO_3_:MoS_2_.

µ-photoluminescence (µ-PL) spectroscopy was employed to investigate the emission properties of MoS_2_ flakes embedded into the AgPO_3_ matrix. Figure 4a presents the steady state PL spectra of all the samples. The red, black, and blue curves correspond to the PL spectra of AgPO_3_, Si/MoS_2_, and AgPO_3_:MoS_2_, respectively. As a reference, we first measured the intrinsic PL spectra of MoS_2_ flakes deposited on Si substrates, using excitation energy of 2.28 eV (543 nm); considering that the direct excitonic transition is weakened with increasing the layer number in MoS_2_, a broad emission peak with weak intensity was observed (Fig. S4)^18,39^. It is notable that the MoS_2_ spectrum has been dramatically changed upon its incorporation in AgPO_3_. Indeed, the spectrum exhibited two well-defined emission peaks at 1.89 and 2.05 eV, corresponding to A- and B- excitonic transition of MoS_2_, respectively. At the same time the PL emission is significantly enhanced, corresponding to a 5- and 6-fold enhancement in the A- and B-exciton peak intensities (Fig. 4b). It should be noted that the PL spectrum of AgPO_3_ glass, presented in Fig. 4a shows no emission within the MoS_2_ excitonic emission range. Notably, besides the enhancement in the PL intensity, and contrary to the conventional PL properties of few-layered MoS_2_, a dominant B-excitonic peak is observed in the AgPO_3_:MoS_2_ emission spectra (Fig. 4b). The corresponding intensity ratio of B- and A-excitons (I_B_/I_A_) equals to 1.2.

To understand the effect of AgPO_3_ matrix on the PL properties, we have investigated the internal structure of AgPO_3_ by means of TEM microscopy. TEM studies reveal a bimodal size distribution of Ag nanoparticles with dominant average sizes of 8.4 nm and 14.5 nm while a broad size variation was also observed (Fig. S5c). The elemental composition of Ag was confirmed by EDX mapping (Fig. S5c). In this context, the large enhancement of MoS_2_ PL intensity observed in the AgPO_3_:MoS_2_ system can be attributed to the localized surface plasmon effect due to the presence of Ag NPs. In order to provide concrete evidence that the observed enhancement of AgPO_3_:MoS_2_ PL intensity is induced by the presence of surface plasmon of Ag particles, we prepare a similar NaPO_3_:MoS_2_ heterojunction, i.e. in which the silver is replaced by sodium, while the phosphate glass network remains unchanged.

Figure 4c shows that the steady state absorption spectra of NaPO_3_:MoS_2_ glass exhibits the two characteristic features at 1.84 and 2.03 eV, which are attributed to intrinsic A- and B-excitonic peaks of MoS_2_, respectively. Moreover, Raman spectroscopy reveals the presence of a few MoS_2_ layers within the fabricated NaPO_3_:MoS_2_ composite glass (Fig. S6). The NaPO_3_ glass exhibits its own characteristic vibrational modes at around 1155 cm^−1^ and ~ 681 cm^−1^, respectively. Contrary to the case of AgPO_3_:MoS_2_, no enhancement of the MoS_2_ PL is found for the NaPO_3_:MoS_2_ system. Indeed, the corresponding room temperature PL spectrum of NaPO_3_:MoS_2_ (Fig. 4d), displays only a very broad and extremely weak emission in the range of the direct A- and B-excitonic transitions. It is therefore clear that the remarkable enhancement on the emission properties of the AgPO_3_:MoS_2_ is induced by the presence of Ag NPs and their plasmon resonance.

The surface plasmon resonance ($\omega_{LSPR}$), which can be tuned by varying the size and shape of the nanostructures and surrounding dielectric medium, is known to strongly modify the excitonic emission^25^. In particular, the plasmon resonance was tuned by changing the nanostructure size, which enhanced the intrinsically weakly emitting B exciton of a MoS_2_ flake^25^. We investigated how the silver plasmon resonance affects the emission properties of the developed MoS_2_ glass heterojunctions upon changing silver content and particle size in the glass. To this aim, an additional glass-MoS_2_ heterojunction was fabricated upon employing the ternary silver-rich 0.3AgI–0.7AgPO_3_ glass instead of the binary AgPO_3_ glass, i.e. for the development of 0.3AgI–0.7AgPO_3_:MoS_2_ architecture. In one of our previous studies it was demonstrated that the incorporation of AgI in the AgPO_3_ glass results to the agglomeration of silver nanoparticles for the formation of larger silver phases^26^, while the phosphate network connectivity remains unaffected. Namely, it was reported that for the aforementioned nominal glass composition silver clusters (larger particles formed from the agglomeration of many nanoparticles) with an average size of 2.78 μm are formed while randomly positioned along the glass network (Fig. S7). The absorption spectrum of bare 0.3AgI–0.7AgPO_3_ exhibited the broad feature of absorption with a hump at ~ 2.47 eV (Fig. S7).

**Figure 5:**
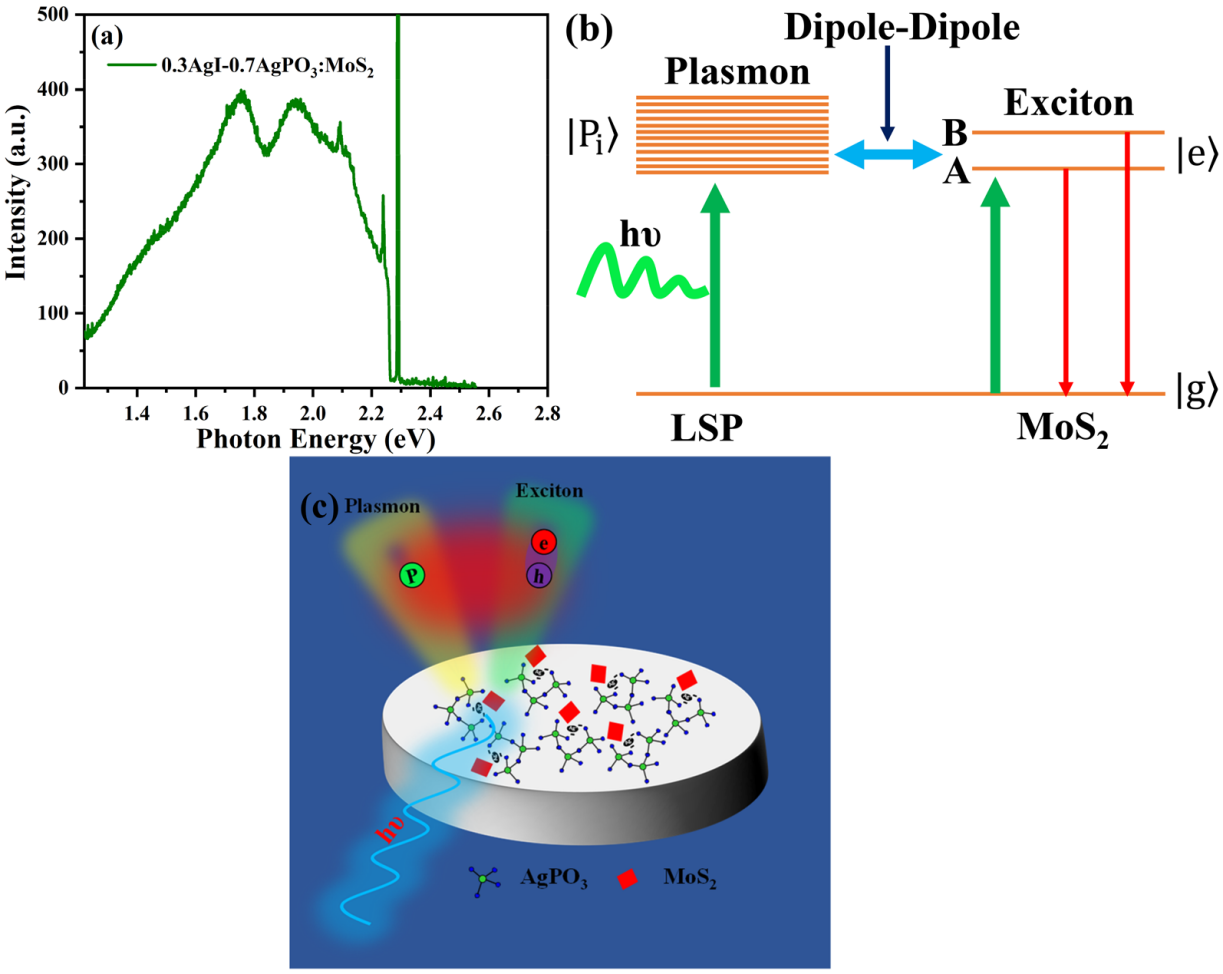
(**a**) Room temperature PL emission spectrum of 0.3AgI-0.7AgPO_3_:MoS_2_; Schematic (**b**) of the mechanism illustrating the exciton–plasmon coupling (**c**) in MoS_2_ and AgPO_3_ via dipole–dipole interaction.

We now consider the effect of these large silver phases on the exciton emission properties of the so-formed 0.3AgI–0.7AgPO_3_:MoS_2_ heterojunctions. The measurement conditions were kept identical to these employed for the AgPO_3_:MoS_2_ measurements. However, the MoS_2_ emission spectrum has changed upon its incorporation in 0.3AgI–0.7AgPO_3_ when compared to AgPO_3_ (Fig. 5a). Specifically, the PL spectrum exhibits two well defined excitonic emission peaks at 1.75 eV and 1.94 eV, corresponding to A- and B-excitonic transitions of MoS_2,_ respectively. The obtained shift in B-exciton peak position has been appeared to be 110 meV. These excitonic peaks are red shifted when compared to the considerably smaller size Ag NPs of the AgPO_3_:MoS_2_ architecture, and their intensities are almost identical. The corresponding intensity ratio of B- and A-excitons (I_B_/I_A_) is 0.99 in this case, compared to 1.2 of AgPO_3_:MoS_2_. Huang et al.^25^ have observed similar red-shifts in the peak position for the A and B excitons, due to the nanocavity resonance controlled by the size of plasmonic nanostructures. Thus, the enhancements in the peak intensities and positions of the excitonic transitions are strongly modified by the plasmon nanostructure size in metaphosphate glass.

### Ultrafast carrier dynamics in MoS_2_ nanoheterojunctions

**Figure 6:**
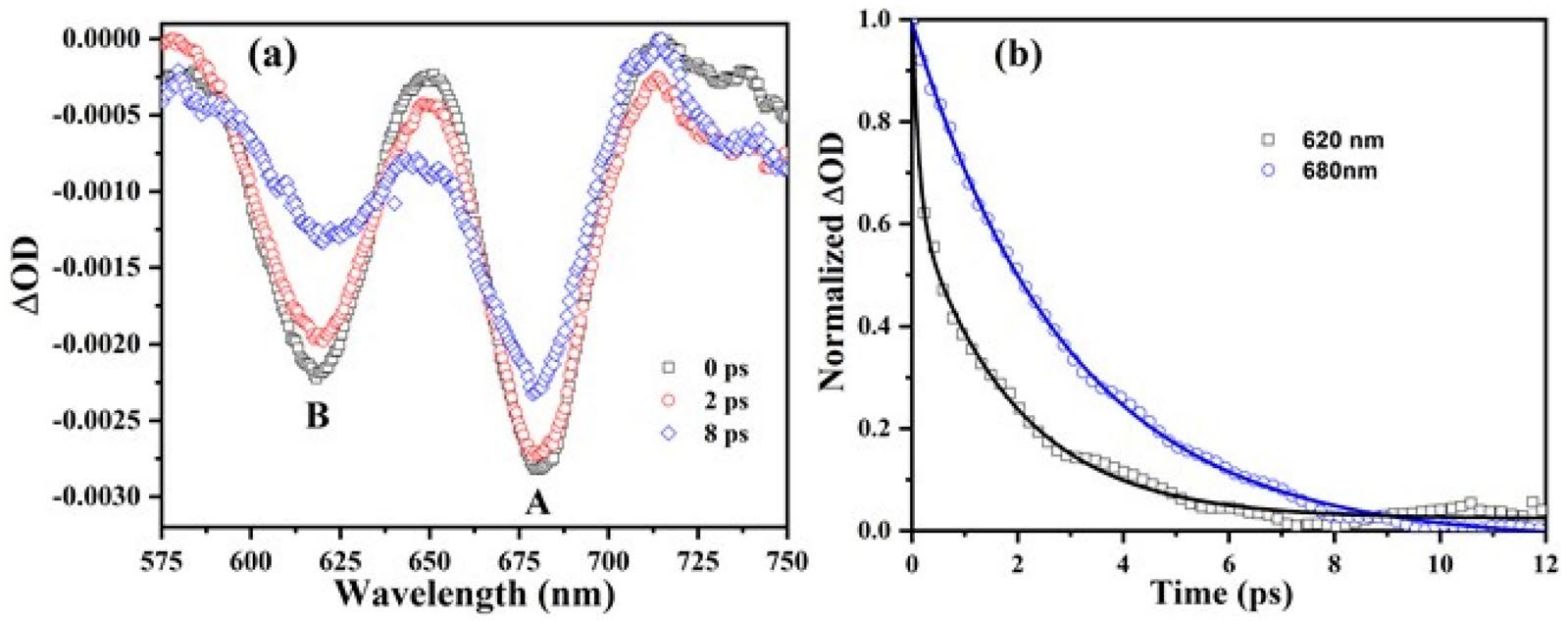
Transient absorption study of AgPO_3_:MoS_2_ nanoheterojunctions. (**a**) Optical density (ΔOD) vs. wavelength plots at various time delays following photoexcitation of AgPO_3_-MoS_2_ glass at 1026 nm with a pump fluence of 2.8 mJ cm^−2^. (**b**) Normalized transient bleach kinetics (symbols) of the A- and B-excitons at 680 and 620 nm, respectively. The solid lines represent the corresponding decay exponential fits.

Finally, in order to further shed light on the observed enhancement in A- and B-excitonic emissions we investigated the corresponding charge carrier relaxation dynamics by means of ultrafast pump probe time-resolved transient absorption spectroscopy (TAS) (Fig. S8)^40,41^. Figure 6a presents optical density (ΔOD) vs. wavelength plots at various time delays following photo-excitation of the AgPO_3_:MoS_2_ glass using a pump fluence of 2.8 mJ cm^−2^. Figure 6b shows the photo-bleaching recovery kinetics of A- and B- exciton states at 680 nm (1.82 eV) and 620 nm (2 eV), respectively. In agreement to previous findings^42^, it is observed that the formation of the A exciton is around 0.5 ps slower when compared to that of the B exciton. In particular, the maximum photo-bleaching of the latter is obtained instantly upon photo-excitation at almost 0 ps. This finding is attributed to the electron–hole cooling time from the upper valence state to the lower valence state within the valence band of the MoS_2_^42^.

Furthermore, upon following typical exponential fittings, we were able to distinguish the physical mechanisms of A- and B-exciton decay dynamics^42,43^. For the latter exciton state, the bi-exponential fitting procedure based on the equation y = y_o_ + A_1_ exp(− x/τ_1_) + A_2_ exp(− x/τ_2_), clearly reveals the presence of two distinct times. Namely, an ultrafast component (τ_1_) of around 0.5 ps that corresponds to electron transfer from the MoS_2_ exciton to AgPO_3_, and a slightly slower time component (τ_2_) of around 2 ps that is attributed to carrier-carrier interactions^42,43^. Rather differently, in the case of A-exciton the ultrafast time component is apparently absent, a finding that implies no electron transfer from the lower energy excitation state towards the metallic particles of the hosting glass. The fast charge transfer present only in the B-exciton, explains why the PL enhancement for the B-exciton is only sixfold and comparable to the five-fold observed for the A-exciton (Fig. 4b). There are two effects taking place in the B-exciton during the photoexcitation process (i) a PL enhancement due to the efficient dipole coupling of exciton–plasmon and (ii) a fast charge transfer from the MoS_2_ to the AgPO_3_. These effects are antagonistic and lead to the observed enhancement.

Based on the aforementioned results, the plasmon coupling in silver based glasses and MoS_2_ heterojunction can be facilitated by either electromagnetic field enhancement due to localized surface plasmon (LSP) effect in Ag NPs, and/or via efficient charge injection between the Ag NPs and MoS_2_ flakes^18,44,45^. Screening and scattering effects due to the presence of metallic NPs could also slightly influence the PL intensities^18^. Moreover, heating and strain effects induced by the glass matrix could also contribute to the change in the PL spectrum observed^39,46,47^. However, such effects should have negligible influence on the PL enhancement in our case due to the inherent indirect band gap^18^, coupled with large thermal conductivity^48,49^ of the few-layered MoS_2_ flakes. It can thus be concluded that the exciton (in MoS_2_)-plasmon (in Ag) coupling (or LSP) is the most plausible explanation for the observed enhancement in PL intensity in Ag based heterojunctions.

To this date, the investigation of surface plasmon induced PL enhancement is only reported in the case of monolayer TMDs^24,50^. In particular, it is observed that the exciton–plasmon coupling is greatly influenced by the contact area between the plasmon nanostructure and 2D material^9,47,51,52^. In our case, it is obvious that the AgPO_3_ glass comprises plenty of nanoheterojunctions among MoS_2_ and Ag NPs, than in the 0.3AgI–0.7AgPO_3_ glass. The AgPO_3_ glass contained smaller diameter nanostructures than 0.3AgI–0.7AgPO_3_ (2.78 μm nanocluster), which can significantly enlarge the contact area and the spatial distribution of the localized electromagnetic field. Besides this, the exact modification of the A- and B- excitonic peaks should strongly depend on the nanoheterojunctions cavity resonance (dipole–dipole interaction), which is controlled by nanostructure size^24^. The steady state photoluminescence enhancement (η) is explained by the change in the quantum yield of the MoS_2_ in the presence of plasmonic nanostructures. The PL quantum yield Y is defined by^53^

$$ {\text{Y}} = \left( {\frac{{k_{r} }}{{k_{r} + k_{nr} }}} \right) $$

where, $k_{r}$ and $k_{nr}$ is the radiative and the nonradiative decay rates. The radiative decay rate is affected by the localized surface plasmonic fields whereas the nonradiative decay rate depends on plasmonic losses and exciton quenching.

The significant PL enhancement in both Ag—glass based nanoheterojunctions indicates an effective coupling between MoS_2_ excitons and LSP resonances in nanostructures with large increase in the radiative decay rate. The plasmonic absorption transition dipole moment which is the collective oscillations of the surface electrons in AgPO_3_ nanostructures that interacts with the transition dipole moments of MoS_2_ (excitonic states of A and B) leading to collective states (Fig. 5b). Such states are often called hybrid states and result in stronger optical PL than the isolated states of the TMD. Since the plasmonic absorption band of 2.0 eV in AgPO_3_ (there are two bands, one at 2.0 eV and the other at 2.5 eV) is in the vicinity of B- exciton transition of MoS_2_ (2 eV) there is a higher probability for B-exciton plasmon dipole–dipole interaction due to the local field enhancement. The physical mechanism behind this process is illustrated in Fig. 5b, c. Altogether, the appearance in discrete and enhanced excitonic emission is led by the exciton (MoS_2_) and surface plasmon (glass) coupling in the nanoheterojunctions (Glass:MoS_2_) system. Further work is currently in progress to optimize this coupling via tuning of the Ag NPs size and fraction^26^ into the AgPO_3_ matrix.

## Conclusion

We have fabricated and demonstrated novel hybrid nanoscale heterojunctions of layered MoS_2_ and metaphosphate glasses. The MoS_2_ phase integrity and excitonic bands are preserved inside the glasses. The developed AgPO_3_:MoS_2_ composite heterojunctions exhibit a remarkably enhanced PL intensity with the presence of well-defined excitonic transitions. A strong modification of A- and B- exciton peak intensity by plasmonic nanostructure has been adopted. We have obtained a six-fold enhancement factor for the intrinsically weak B exciton peak. Such enhancement factor for the B excitonic emission is explained with the help of dipole–dipole interaction via exciton–plasmon coupling. The ultrafast electron transfer process and carrier-carrier interaction in the nanoheterojunction system support the enhancement in the B excitonic emission. No doubt, the efficient dipole coupling of exciton–plasmon and tunability of B- excitonic emission find application in emerging valleytronic devices working with B excitons. Moreover, the presented fabrication process might be promising for large scale production of inexpensive nanophotonic, valleytronics and optoelectronic devices with tunable B excitonic emissions.

## Methods

### Sample preparation

#### MoS_2_ nanoflakes

MoS_2_ flakes were prepared from bulk MoS_2_ powder (grain size < 2 µm, Sigma Aldrich) using liquid phase exfoliation (LPE) method, as reported elsewhere^54,55^. In detail, 40 mg of bulk MoS_2_ powder was dissolved in 10 ml IPA. The solution was ultrasonicated for 60 min in a Elma S 30 H bath sonicator (Elma Schmidbauer GmbH, Germany) under 80 W power and 37 kHz frequency. Room temperature (< 30 °C) was maintained throughout the exfoliation process (bath sonicator). After ultrasonication the dispersion was centrifuged to exclude the unexfoliated bulk MoS_2_. The supernatant of the resulting dispersion was collected and used for subsequent experiments.

#### AgPO_3_ glass and AgPO_3_:MoS_2_ nanoscale heterojunction formation

The development of AgPO_3_:MoS_2_ heterojunction glasses relies on the incorporation of MoS_2_ flakes within silver metaphosphate glass (AgPO_3_). First, a previously described procedure was followed for the preparation of the AgPO_3_ glass substrates^37,38^. Namely, equimolar amounts of high-purity AgNO_3_ (99.995%) and NH_4_H_2_PO_4_ (99.999%) dry-powders were melted in a platinum crucible. All weighing and mixing manipulations of the two powders were performed within a glove bag purged with dry nitrogen gas. After thorough mixing of the two powders, the melting batch was transferred to an electrical furnace initially held at 170 °C, while slowly heated up to 290 °C for the smooth removal of the volatile gas products. The furnace temperature was then raised to 450 °C and kept steady for 30 min, while performing frequent stirring in order to ensure melt homogeneity. AgPO_3_ glasses were obtained in the form of 1 mm thick disk specimens with a diameter of around 10 mm, upon splat-quenching the melt. This well-established procedure results in AgPO_3_ glasses with negligible water traces of less than 0.3 mol%, i.e. incapable of causing any optical or structural property modifications. Moreover, the glasses remain unaffected of room humidity (25–30%) for several months.

For the incorporation of MoS_2_, the AgPO_3_ glass substrate was positioned on a silicon wafer while a heating plate was employed in order to maintain a temperature around 80 °C. Ten drops of a previously prepared MoS_2_ solution (0.76 mg/ml) were drop-casted on the surface of the AgPO_3_ glass, while allowing 10 s intervals between each drop in order to ensure smooth solvent vaporization. After solvent removal the residual MoS_2_ flakes were randomly distributed on the AgPO_3_ surface. Then, the temperature was raised to 170 °C for 2 min, i.e. 22 °C below the glass transition temperature of the AgPO_3_ glass. At this temperature, the AgPO_3_ glass becomes viscous and allows readily the smooth incorporation of the MoS_2_ flakes within the glass matrix. Following MoS_2_ immersion, the AgPO_3_:MoS_2_ nano-hybrid glass was splat-quenched between two silicon wafers, while instantly removed from the heating plate and left to cool down to room temperature. The employment of silicon wafers allows the formation of smooth surfaces on both sides of the composite glass specimens and renders them suitable for optical characterization. The MoS_2_ incorporation process is presented in Schematic S1, while the samples used are presented in Table 1.

**Table 1:** Description of the samples used in this study.

Sample	Description
Bare MoS_2_	Isolated from bulk powder
AgPO_3_	Silver metaphosphate glass
Si/MoS_2_	MoS_2_ on Si substrate
AgPO_3_/MoS_2_	MoS_2_ on the top of AgPO_3_
AgPO_3_:MoS_2_	MoS_2_ embedded into AgPO_3_
NaPO_3_	Sodium metaphosphate glass
NaPO_3_:MoS_2_	MoS_2_ embedded into NaPO_3_
0.3AgI-0.7AgPO_3_:MoS_2_	MoS_2_ embedded into 0.3AgI-0.7AgPO_3_

### Optical measurements

The optical UV–Vis absorption spectra of the dispersion and solid films were carried out with a PerkinElmer, Lamda 950 UV/VIS/NIR spectrometer, USA. The Raman spectra were recorded under 473 nm laser excitation (Thermo Scientific) in the back-scattering geometry at ambient conditions at 300 K. The Si substrate peak at 520 cm^−1^ was used for calibration purposes.

For optical spectroscopy measurements, we used a Micro-Photoluminescence (μ-PL) setup and the spectra were collected in a backscattering geometry at 300 K. As excitation source was used a continuous wave (CW) He–Ne 543 nm (2.28 eV) laser. An iHR-320 spectrometer (Horiba Scientific/Jobin Yvon Technology) equipped with a Syncerity multichannel charge-coupled device (CCD) Camera was employed to collect the spectra.

For the XRD measurements an X-Ray Rigaku (D/max-2000) diffractometer was employed, while being operated with a continuous scan of Cu Ka1 radiation with λ equal to 1.54056 Å. The morphology of the Ag NPs was studied by transmission electron microscopy (TEM, LaB6 JEOL 2100), after depositing drops of glass-powder/toluene solution onto a carbon-coated TEM grid. Finally, a field emission scanning electron microscope (SEM, JEOL, JSM-7000F) was used for the examination of the lateral dimension of dispersed 2D MoS_2_ flakes, while atomic force microscopy (AFM) was employed to obtain the flakes’ thickness (Digital Instruments with controller Nanoscope IIIa).

## Supplementary information

Supplementary file1
