# Supplementary material for: Robust B-exciton emission at room temperature in few-layers of MoS2:Ag nanoheterojunctions embedded into a glass matrix
Source: Sci Rep. 2020 Sep 24;10:15697. doi: 10.1038/s41598-020-72899-3 (PMC7518262; doi:10.1038/s41598-020-72899-3)
Supplement: Supplementary file 1 — Supplementary file1 [file 41598_2020_72899_MOESM1_ESM.docx]

**Supplementary Information**

**Robust B-exciton emission at room temperature in few-layers of MoS_2_:Ag nanoheterojunctions embedded into a glass matrix**

Abdus Salam Sarkar,^1*^ Ioannis Konidakis,^1^ [Ioanna Demeridou](http://stratakislab.iesl.forth.gr/team/ioanna-demeridou/),^1,2^ Efthymis Serpetzoglou,^1,2^ George Kioseoglou,^1,3^ and Emmanuel Stratakis^1,2*^

^1^Institute of Electronic Structure and Laser, Foundation for Research and Technology-Hellas, Heraklion, 700 13 Crete, Greece.

^2^Physics Department, University of Crete, Heraklion, 710 03 Crete, Greece.

^3^Department of Materials Science and Technology, University of Crete, Heraklion, 710 03 Crete, Greece.

**Email**: [salam@iesl.forth.gr](mailto:salam@iesl.forth.gr); [stratak@iesl.forth.gr](mailto:stratak@iesl.forth.gr)

**μ-PL set up**

In the μ-PL set up the laser beam passes a short pass (SP) filter (FESH550, Thorlabs) to reduce the noise at higher wavelengths. The power of the laser beams is controlled via a neutral density (ND) filter (Thorlabs). A 50:50 beam splitter (BS 50:50, Thorlabs) is used to reflect and drive the beam to the objective lens. A Mitutoyo 50x (NA:0.42, *f*=200 mm) focuses down to ~1 μm the spot size for the sample excitation. The position of the sample is controlled with a XYZ mechanical translation stage (PT3, Thorlabs). Following the excitation, the emitted PL signal passes through a long pass (LP) filter (FELH550, Thorlabs) to eliminate the emission of the laser.


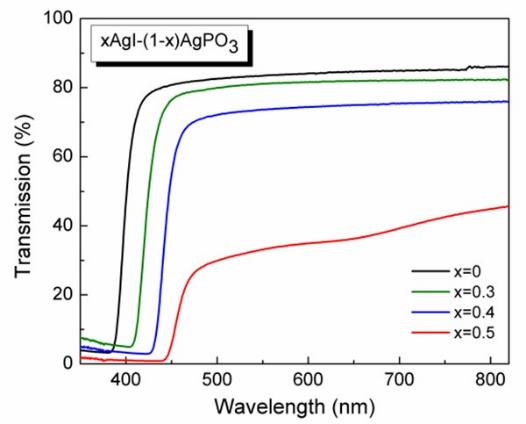


**Fig. S1** Transmission spectra of xAgI-(1-x)AgPO_3_ glasses.


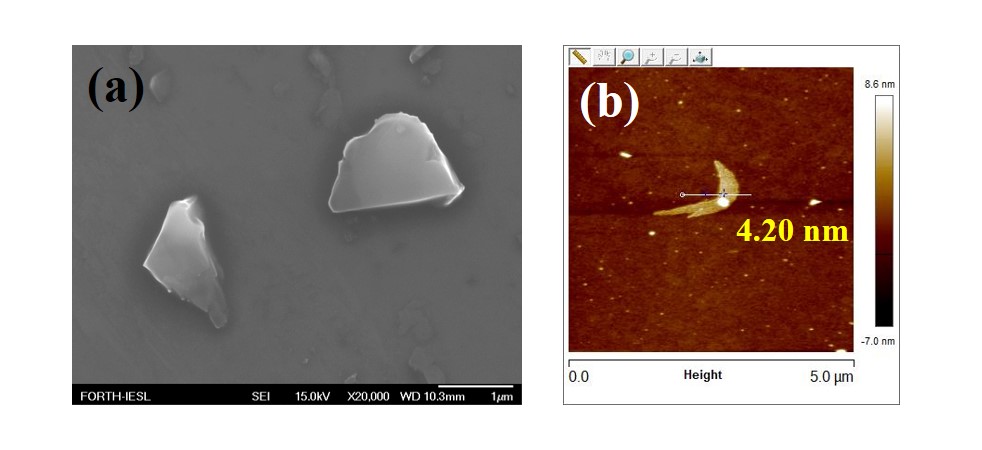

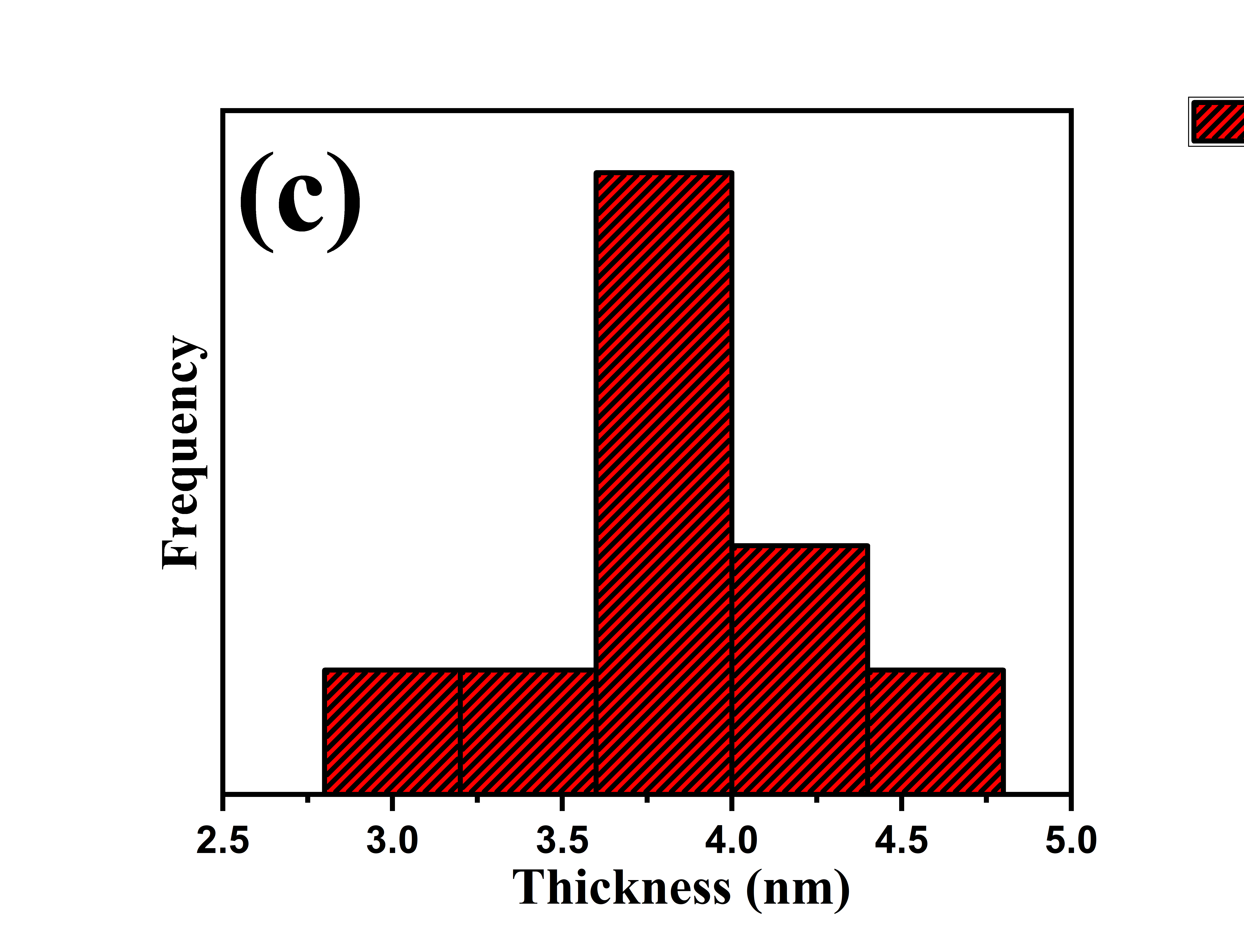


**Fig. S2** Microscopic images of MoS_2_ (a) SEM micrograph of MoS_2_ flakes deposited on Si substrates and (b) AFM image with height profile (along solid line). Scale bar is 5 μm. and (c) histogram of thickness distribution.


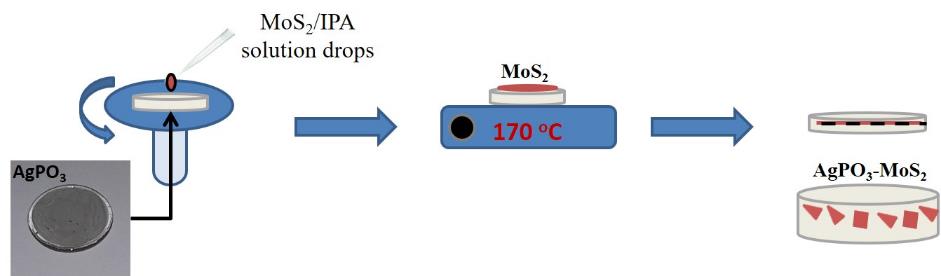


**Schematic S1**. The schematic representation of the incorporation of two dimensional MoS_2_ inside silver metaphosphate glass matrix. First left panel: optical image of the glass matrix.


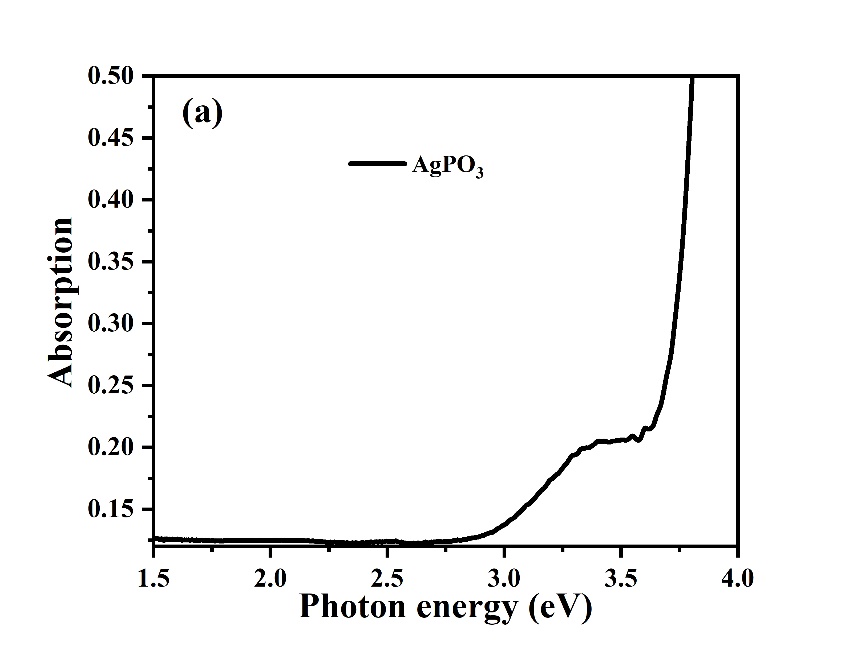

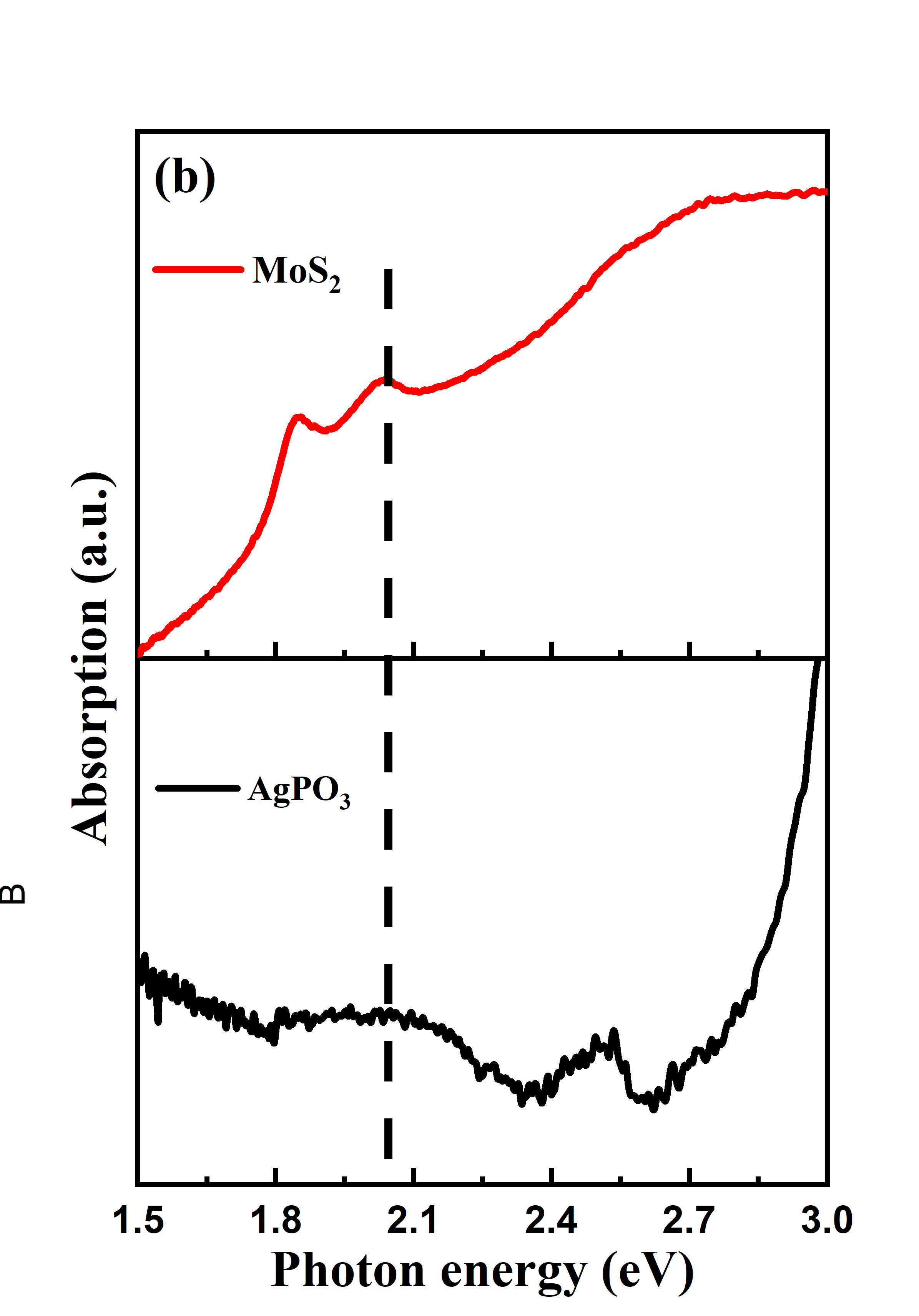


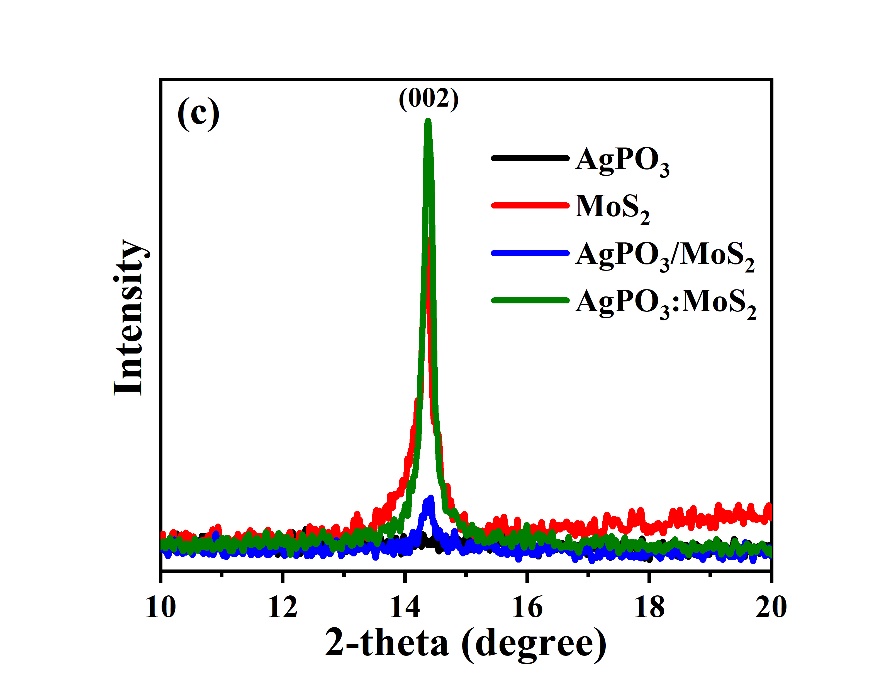

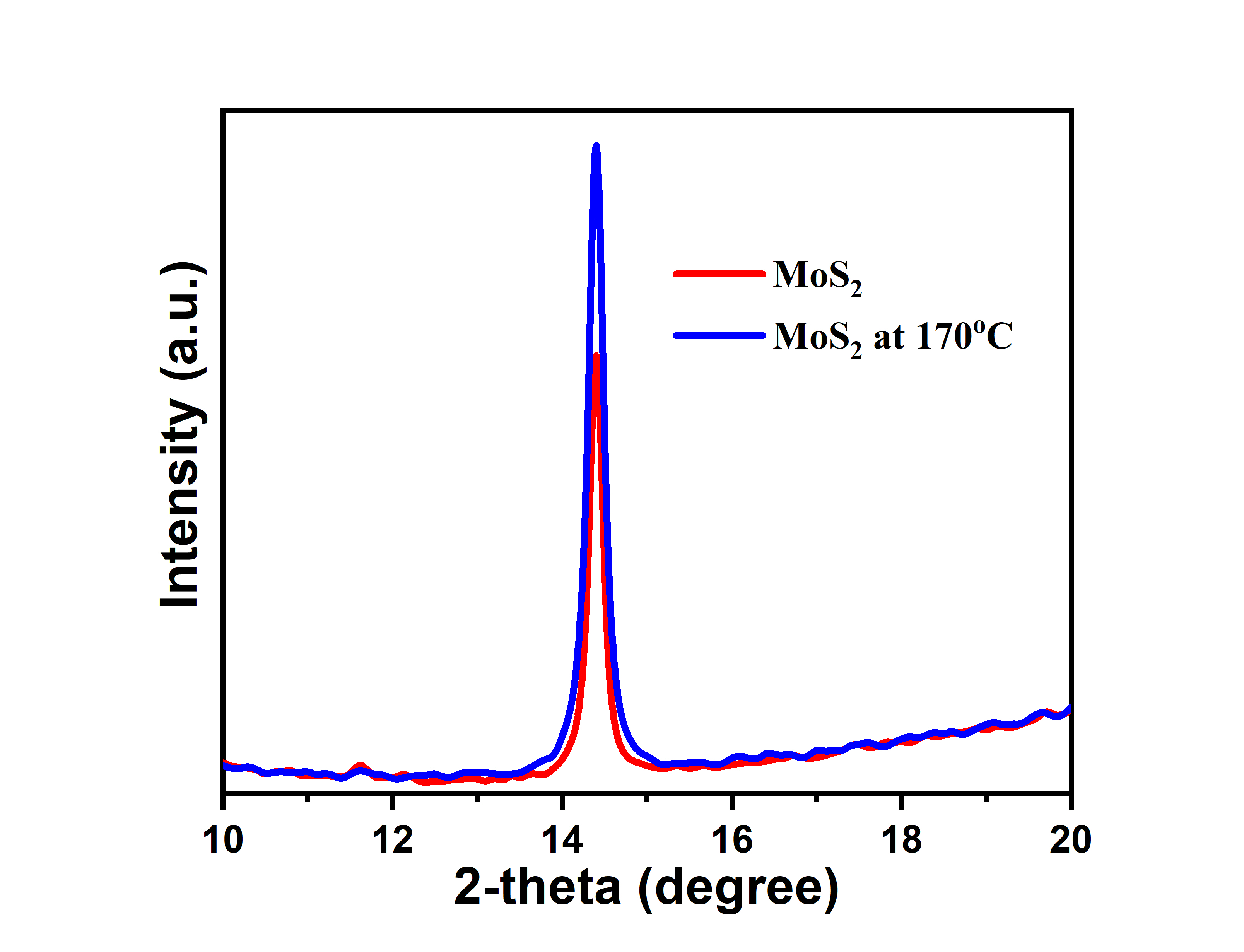


**(d)**

**Fig. S3** (a) Optical absorption spectra of silver metaphosphate glass (AgPO_3_) matrix (left panel), (b) zoomed absorption spectra of MoS_2_ and AgPO_3_, (c) The magnified hexagonal XRD peak at ~14º (002 plane) of MoS_2_ and heterojunctions, and (d) XRD pattern of controlled MoS_2_ and MoS2 treated with 170^o^C.

**Table S1.** X-ray diffraction data fitting parameters (peak positions and full width at half maximum)

| **Sample** | **Peak position (2θ)**  **in degree** | **FWHM** |
| --- | --- | --- |
| MoS_2_ | 14.34 | 0.367 |
| AgPO_3_/MoS_2_ | 14.38 | 0.317 |
| AgPO_3_:MoS_2_ | 14.38 | 0.225 |


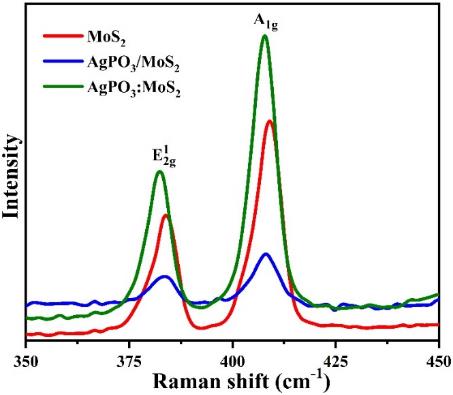

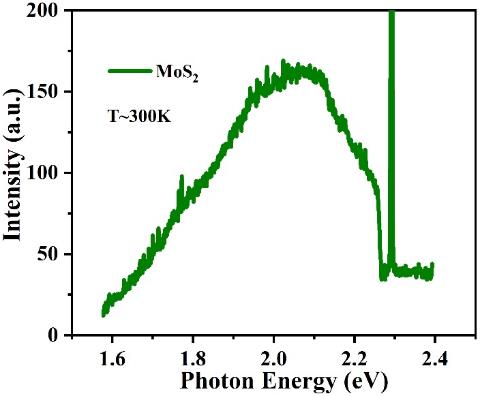


**Fig. S4** Left panel: Raman spectra of MoS_2_ (MoS_2_ on Si), AgPO_3_/MoS_2_ (MoS_2_ on AgPO_3_ glass), and AgPO_3_:MoS_2_ (MoS_2_ in AgPO_3_ glass matrix) or nanoheterojunctions. Right panel: PL spectra of MoS_2_ on Si substrate.


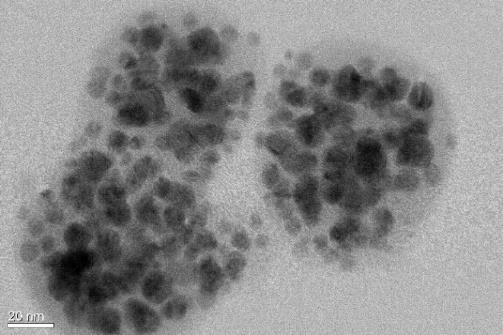

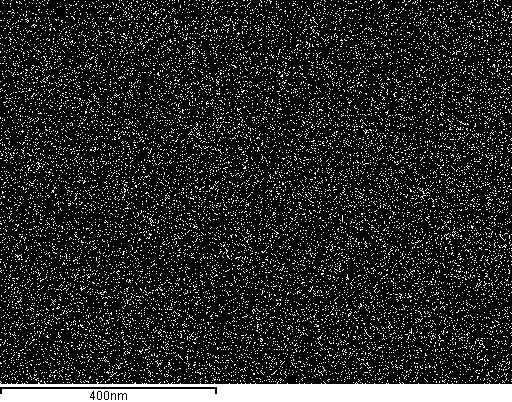


**(b)**

**(a)**


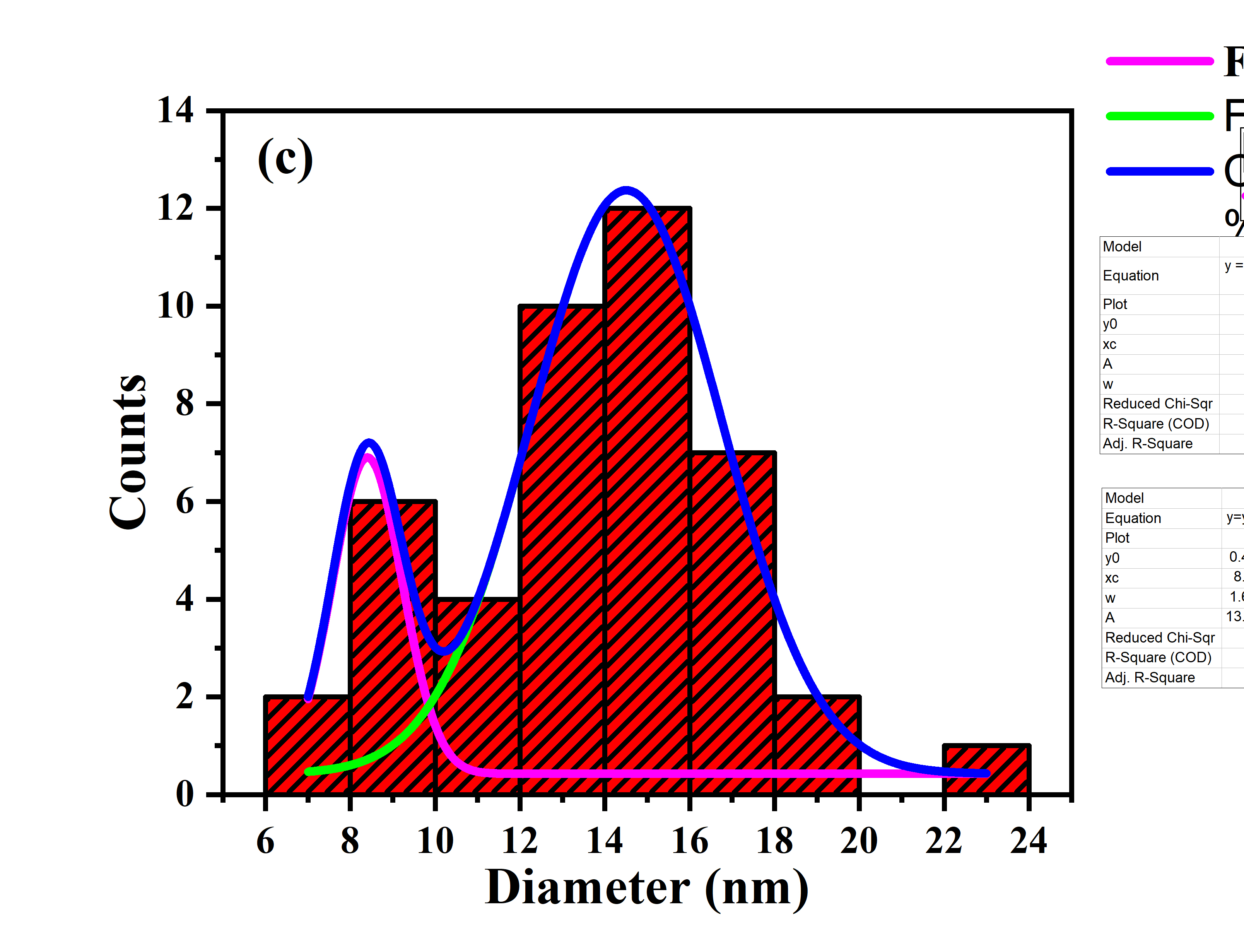


**Fig. S5** (a) TEM micrograph of AgPO_3_ glass and (b) Energy Dispersive X-Ray Analysis of Ag nanostructures and (c) Histogram of nanostructure size distribution. Blue, green and pink lines are gaussian fitting. Average sizes of nanostructure is ~8.4 and 14.5 nm.


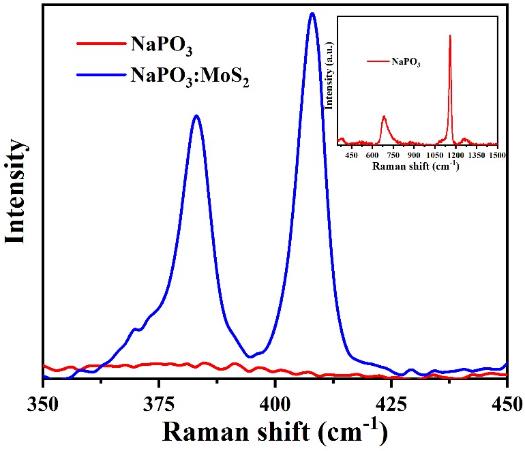


**Fig. S6** Room temperature Raman spectra of NaPO_3_ glass and NaPO_3_-MoS_2_ heterojunctions. Inset: NaPO_3_ Raman spectra in wide range.

**(a)**


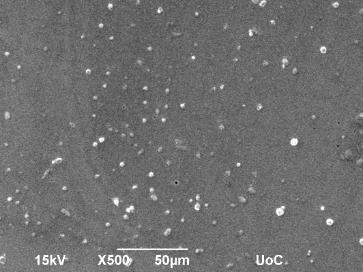

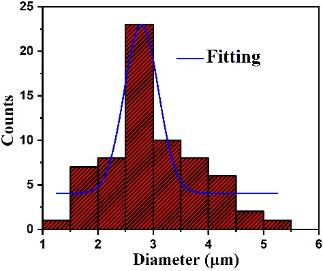


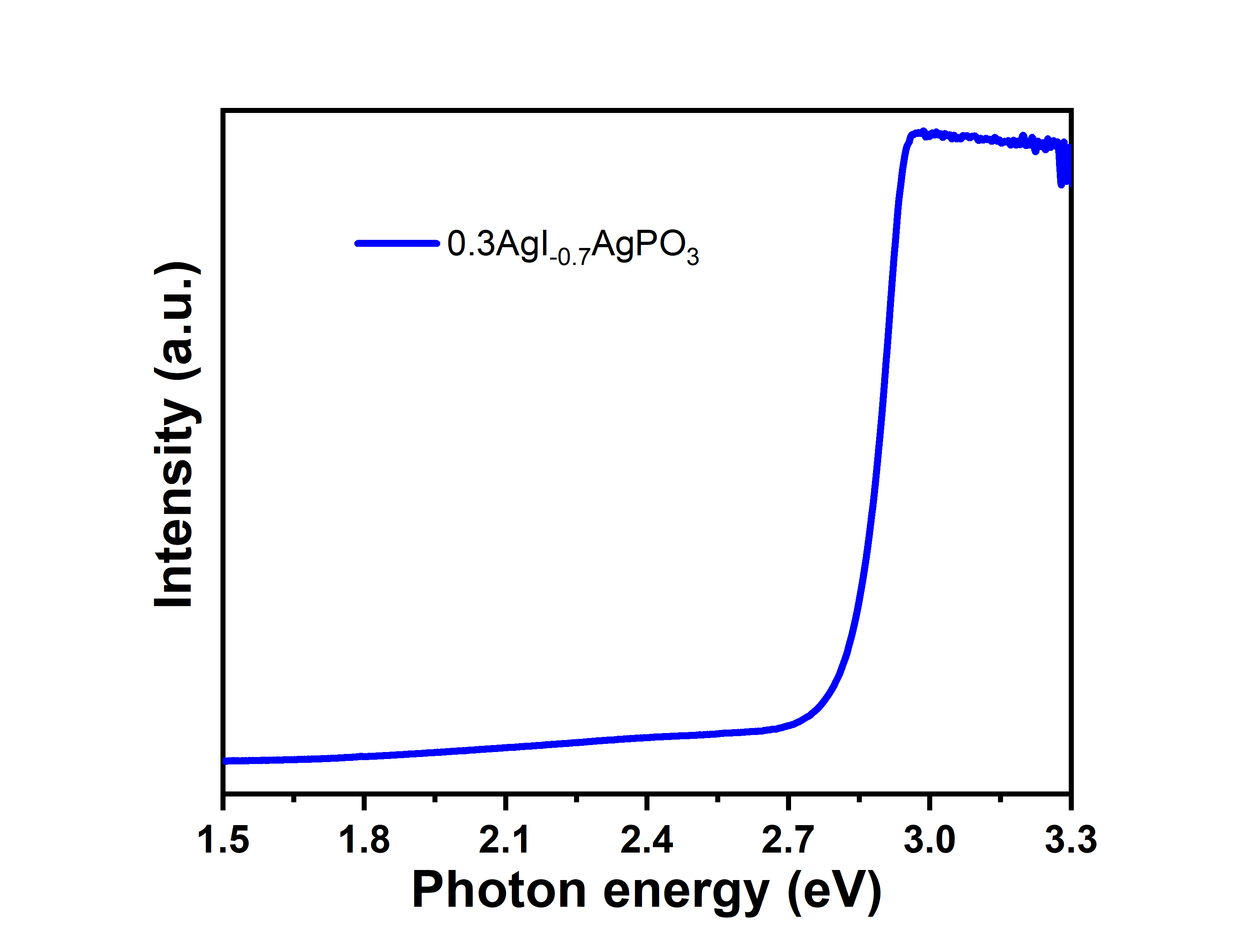

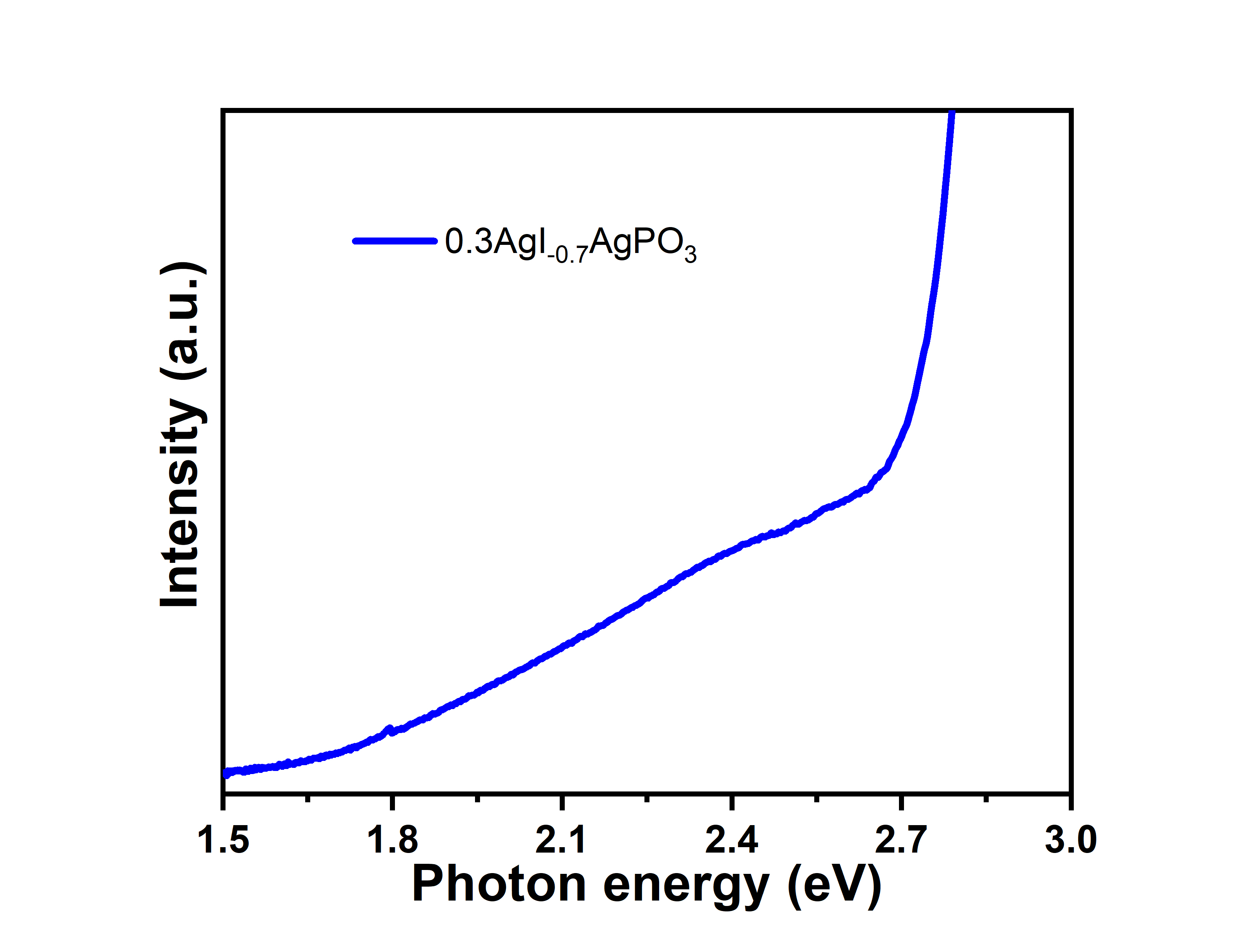


**(b)**

**Fig. S7** (a) FESEM image and histogram of Ag nanostructure size distribution of 0.3AgI-0.7AgPO_3_ glass. Blue line is gaussian fitting. Average size of nanostructure is ~2.78 μm).

**AgPO_3_-MoS_2_ TAS experimental**

Pump-probe transient absorption spectroscopy (TAS) measurements were performed on a Newport (TAS-1) transient absorption spectrometer, equipped with a source pulsed laser beam generated from an Yb:KGW-based laser system (PHAROS, Light Conversion), emitting at 1026 nm, with a pulse duration of 170 fs and 1 KHz repetition rate. As shown schematically in **Fig. S8** the 1026 nm fundamental beam was split, so that the probe beam component (10% of the source) passes through a delay line and routed on a YAG crystal, which generates a supercontinuum white light of 500-920 nm. The other part of the incident beam (90% of the source), was used as the pump beam for sample excitation. The energy of the pump beam was controlled by a variable reflective neutral density filter inside the TAS instrument. The probe light was coupled through an optical fiber to a multichannel detector and monitored as a function of wavelength. In a typical TAS pump-probe experiment, the sample is excited by the pump beam, and the corresponding decay dynamics of the sample’s relative optical density are recorded as a function of wavelength at various time delays after photo-excitation. Measurements of the present study were performed on 1 mm thick AgPO_3_-MoS_2_ composite glass specimens, with a pump beam fluence of 2.8 mJ cm^-2^.


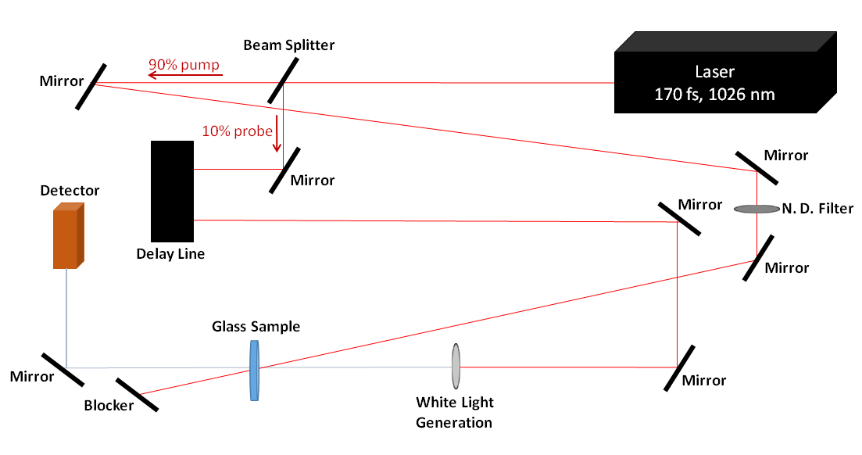


**Fig. S8.** Schematic representation of transient absorption spectroscopy (TAS) experimental setup (Newport, TAS-1).
